# Antimicrobial Use, Human Gut Microbiota and *Clostridium difficile* Colonization and Infection

**DOI:** 10.3390/antibiotics4030230

**Published:** 2015-07-03

**Authors:** Caroline Vincent, Amee R. Manges

**Affiliations:** 1 Department of Microbiology and Immunology, McGill University, Montréal, QC H3A 2B4, Canada; E-Mail: caroline.vincent3@mail.mcgill.ca; 2 School of Population and Public Health, University of British Columbia, Vancouver, BC V6T 1Z3, Canada

**Keywords:** *Clostridium difficile* infection, intestinal microbiota, antimicrobials, colonization resistance, fecal microbiota transplantation

## Abstract

*Clostridium difficile* infection (CDI) is the most important cause of nosocomial diarrhea. Broad-spectrum antimicrobials have profound detrimental effects on the structure and diversity of the indigenous intestinal microbiota. These alterations often impair colonization resistance, allowing the establishment and proliferation of *C. difficile* in the gut. Studies involving animal models have begun to decipher the precise mechanisms by which the intestinal microbiota mediates colonization resistance against *C. difficile* and numerous investigations have described gut microbiota alterations associated with *C. difficile* colonization or infection in human subjects. Fecal microbiota transplantation (FMT) is a highly effective approach for the treatment of recurrent CDI that allows the restoration of a healthy intestinal ecosystem via infusion of fecal material from a healthy donor. The recovery of the intestinal microbiota after FMT has been examined in a few reports and work is being done to develop custom bacterial community preparations that could be used as a replacement for fecal material.

## 1. Introduction

*Clostridium difficile* infection (CDI) is the leading cause of infectious diarrhea in hospitalized patients and is associated with significant morbidity and mortality. In the United States alone, there are an estimated 453,000 cases and 29,300 deaths from CDI each year [1]. *C. difficile* is a Gram-positive, anaerobic, spore-forming and toxin-producing bacillus that lives in the environment (soil, water), as well as in the gut of animals (cats, dogs, horses, cattle, pigs, poultry) and humans. The infection is associated with a wide range of clinical manifestations, from asymptomatic colonization to mild diarrhea or more severe pseudomembranous colitis that may progress to toxic megacolon, intestinal perforation, sepsis and death [2]. Recognized risk factors for CDI include advanced age, concurrent diseases, previous or prolonged hospital stay (which increases the likelihood of exposure to *C. difficile* spores), gastrointestinal surgery, nasogastric tube feeding, use of proton-pump inhibitors and antibiotic exposure, especially to broad-spectrum agents [2,3]. Patients are exposed to *C. difficile* spores through contact with the hospital environment and will typically become vulnerable to CDI during or after antimicrobial treatment. Broad-spectrum antimicrobials have profound detrimental effects on the structure and diversity of the indigenous intestinal microbiota [4,5]. These alterations often impair colonization resistance, thereby allowing the establishment and proliferation of *C. difficile* in the gut. Although nearly all classes of antibiotics have been associated with CDI, clindamycin, penicillins, cephalosporins, and, more recently, fluoroquinolones seem to pose the greatest risk [6,7,8]. Even though the term “infection” is sometimes used to designate people who are asymptomatically colonized with a pathogen, in this review we will use the term “infection” (or CDI) to describe patients who are symptomatic, and “colonization” to describe patients who are asymptomatically colonized with *C. difficile*.

Among patients who acquire *C. difficile* in their gut, some may go on to develop diarrhea or more severe forms of CDI, while others will become asymptomatically colonized. Differences in pathogen or host factors like the immune status or the integrity of the intestinal microbiota may affect the clinical presentation of CDI. Not all strains of *C. difficile* are toxin-producing. Patients can be asymptomatically colonized with toxigenic or non-toxigenic *C. difficile* strains. However, the acquisition of a toxigenic strain is essential for the development of a symptomatic infection [9]. It has been suggested that patients with asymptomatic *C. difficile* colonization are at decreased risk of developing CDI, but a recent meta-analysis has refuted this hypothesis [10,11]. In hospitals and health-care facilities, asymptomatic carriers often outnumber symptomatic patients and may represent a considerable reservoir of *C. difficile* that contributes to environmental contamination and disease transmission among patients [12,13].

A challenging aspect of CDI is the recurrence of infection after successful completion of therapy and symptom resolution. After an initial episode of CDI, 15%–30% of patients will experience a recurrence of the infection, which typically occurs within four weeks after completion of antibiotic therapy for the initial episode. In patients who have already experienced one recurrence of CDI, the chance of suffering from additional recurrences increases to 40%–65% [14,15,16,17]. Recurrent CDI can turn into a chronic, recalcitrant disease with indefinite cycles of infection relapse leading to persistent use of antibiotics, repeated hospitalizations, complications and even death. A growing body of evidence suggests that persistent alterations of the colonic microbiota are a key factor in the pathophysiology of recurrent CDI. Fecal microbiota transplantation (FMT), which refers to the introduction of fecal material obtained from a healthy individual into the colon of patients suffering from multiply recurrent CDI, is able to halt recurrences, as demonstrated in a recent randomized clinical trial [18]. By re-introducing a healthy gut microbiota, the procedure is thought to restore colonization resistance against *C. difficile* and reestablish a normally functioning intestinal microbiota.

## 2. Impact of Antimicrobial Use on the Human Intestinal Microbiota

Although antibiotics are a life-saving tool for the treatment of bacterial infections, they induce important collateral damage to host-associated microbial communities. Antibiotics have rapid, profound and sometimes long-lasting effects on the structure of the intestinal microbiota [4,19]. Broad-spectrum antibiotics do not only reduce the overall size and diversity of the bacterial population but they also alter the relative proportions of remaining bacterial species as well as the overall functional capacity of the community [20]. As a complex network of co-dependence exists among members of the gut microbiota, the microorganisms that are affected by antibiotics are not necessarily restricted to those that are directly targeted by the antimicrobial agent [21]. Antimicrobial exposure can promote the emergence of resistant microorganisms, which can persistently colonize the gastrointestinal tract even after the selective pressure is removed. The spread of antibiotic resistance genes from commensal to pathogenic bacteria in the gut via horizontal gene transfer is also a concern [19]. The extent of the disturbances to the intestinal microbiota depends on multiple factors: the particular antibiotic used (mode of action, spectrum of activity, degree of absorption, pharmacodynamic and pharmacokinetic properties and *in vivo* inactivation), the dosage and duration of treatment, the route of administration and elimination of the antibiotic, as well as the degree of resistance of the microbial community [19,22]. At a clinical level, antimicrobial-induced intestinal microbiota alterations can result in a diarrheal episode or an opportunistic yeast infection [22]. Antibiotic-associated diarrhea occurs in as many as 15%–25% of hospitalized patients, but usually resolves shortly after treatment discontinuation [23]. An overgrowth of *C. difficile* in the intestines has been observed during or after antimicrobial therapy, and was mainly associated with clindamycin, penicillin, cephalosporin and fluoroquinolone exposure [22,23]. Several culture- and molecular-based studies have evaluated the short- and long-term impacts of these antimicrobial agents on the intestinal microbiota of patients undergoing clinical treatment or healthy volunteers [19,22,23].

### 2.1. Clindamycin

Clindamycin is a broad-spectrum antibiotic that primarily targets anaerobic bacteria as well as Gram-positive aerobic bacteria such as *Staphylococcus aureus* [24]. This antibiotic is mainly excreted in bile and can reach high concentrations in the intestinal lumen, leading to major disruptions of the gut microbiota [19,22]. In a culture-based study of the fecal microbiota of 30 healthy volunteers, clindamycin administration induced a marked decrease in anaerobic bacterial populations (lactobacilli, clostridia, *Bacteroides* and bifidobacteria) and an increase in enterococci and enterobacteria (*Klebsiella*, *Enterobacter* and *Citrobacter*), which are intrinsically resistant to this antibiotic [25]. Another culture-based study looking at the effects of clindamycin prophylaxis in 15 surgical patients observed a temporary decrease of streptococci and enterococci during antibiotic administration [26]. A sharp decrease in anaerobic bacteria also occurred after clindamycin treatment, but the bacterial concentrations returned to their initial levels within two weeks. Jernberg *et al.* used terminal restriction fragment length polymorphism of the 16S rRNA gene to evaluate the long-term impacts of a seven-day clindamycin course on the fecal microbiota of four healthy subjects. A large shift in the composition of the total bacterial community was observed immediately after clindamycin exposure, but the community returned to its original state within three months after treatment. In contrast, the *Bacteroides* never returned to their pre-treatment levels and this disturbance persisted up to two years after clindamycin administration. Dramatic and prolonged increases in the levels of antibiotic resistance genes (*erm*) were also observed in fecal DNA samples after clindamycin exposure [27].

### 2.2. Penicillins

Several studies have examined the impact of amoxicillin (penicillin) or amoxicillin in combination with clavulanate (penicillin with β-lactamase inhibitor) on the human intestinal microbiota. In culture-based investigations, amoxicillin administration with or without clavulanate was associated with an overgrowth of resistant enterobacterial species [22,23]. Barc *et al.* used a human fecal microbiota-associated mouse model to study the effects of a seven-day course of amoxicillin-clavulanate on the intestinal microbiota [28]. Predominant groups of bacteria were quantified by fluorescence *in situ* hybridization and flow cytometry with 16S rRNA oligonucleotide probes. Although the total number of anaerobic bacteria remained stable throughout the experiment, there was a dramatic decrease in the levels of *Clostridium coccoides-Eubacterium rectale* group as well as increases in the levels of *Bacteroides-Porphyromonas-Prevotella* and Enterobacteriaceae groups during antibiotic treatment. All bacterial groups went back to their pre-treatment level within seven days after antibiotic discontinuation. Young and Schmidt monitored temporal changes in the diversity of the fecal microbiota in a patient who developed antibiotic-associated diarrhea during a 10-day treatment with amoxicillin-clavulanate [29]. Fecal samples collected on day 0, 4 and 24 were analyzed by 16S rRNA gene clone libraries. Prior to antibiotic treatment, the fecal microbiota was mainly composed of *Bacteroides*, *Bifidobacterium*, and members of the *Clostridium* cluster IV and XIVa (butyrate-producing bacteria). Four days after treatment initiation, there was a marked increase in members of the Enterobacteriaceae family, but no sequences corresponding to *Bifidobacterium* or *Clostridium* cluster XIVa were detected. Most of these alterations were resolved two weeks after cessation of the antibiotic, except for the *Bifidobacterium* genus, which never recovered. De La Cochetière and co-workers used temporal temperature gradient gel electrophoresis (TGGE) of the 16S rRNA gene to examine the dominant fecal microbiota of six healthy volunteers before, during and after a five-day treatment with amoxicillin [30]. Major shifts in dominant bacterial species were observed three to four days after treatment initiation and the bacterial profiles slowly returned to their original state during the subsequent two months. However, in one subject, fecal microbiota disturbances persisted for at least two months.

### 2.3. Cephalosporins

Because the spectrum of action of cephalosporins is wider than that of penicillins, greater disturbances of the intestinal microbiota can be expected, particularly with agents that are excreted via the biliary duct like ceftriaxone [22]. In culture-based studies, cephalosporin use was shown to decrease the abundance of enterobacteria and increase the levels of aerobic Gram-positive cocci (mainly enterococci, which are intrinsically resistant). This antimicrobial agent is also associated with the emergence of resistance in enterobacteria and overgrowth of yeasts [22,23]. Perez-Cobas and co-workers employed a multi-omic approach to characterize the intestinal microbiome of a patient undergoing cefazolin treatment for 14 days [31]. Fecal samples were collected prior to and during antibiotic treatment, as well as 40 days after treatment. Prior to antibiotic administration and during the first days of treatment, members of the Firmicutes dominated the fecal microbiota. On day 11, there was a collapse in biodiversity that was associated with a displacement of Firmicutes and a remarkable increase in the abundance of Bacteroidetes (*Bacteroides* and *Parabacteroides* genera) and Betaproteobacteria. Based on proteomic and metabolomic analyses, the intestinal microbiota responded quickly (day 6) to antibiotic exposure by activating drug-detoxifying mechanisms (such as the expression of β-lactamases or multidrug efflux pumps), while attenuating the metabolism and transport of bile acids, cholesterol, hormones and vitamins. Forty days after cessation of the antibiotic treatment, the total and active fractions of the intestinal microbiota were similar to the pre-treatment state, suggesting recovery of the original bacterial populations.

### 2.4. Fluoroquinolones

Fluoroquinolones are rapidly absorbed after oral administration and achieve very high concentrations in feces [22]. Enterobacteriaceae are highly susceptible to fluoroquinolones and are therefore strongly suppressed during exposure. Some fluoroquinolones also reduce the number of aerobic Gram-positive cocci [22,23]. Dethlefsen *et al.* tracked changes in the distal gut bacterial communities of three healthy subjects before and after a five-day treatment with ciprofloxacin [4,5]. While the intestinal microbiota of each subject responded uniquely to the antibiotic, some general trends could be observed. The effect of ciprofloxacin on the gut microbiota was profound and rapid, causing an abrupt decline in diversity, eliminating 25%–50% of the bacterial population and producing major shifts in community composition (with reductions in Ruminococcaceae and *Faecalibacterium*) within three or four days of treatment initiation. Although the taxonomic composition of the microbiota closely resembled its pre-treatment state by one to four weeks after the end of the treatment, several bacterial taxa, including Clostridiales and *Bilophila*, had not recovered even six months following treatment [4,5].

## 3. Colonization Resistance against *C. difficile*

Colonization resistance refers to the ability of the microbiota to limit the overgrowth of opportunistic indigenous microorganisms and to prevent the invasion of exogenous, potentially pathogenic microorganisms in the gut. It has been known for a long time that the complete absence of an intestinal microbiota in germ-free mice and the administration of antibiotics to conventional mice or human volunteers greatly enhances the degree of susceptibility to colonization and infection by new microorganisms [32]. The indigenous gut microbiota can mediate colonization resistance directly, via the production of growth-inhibitory or toxic substances (e.g., bacteriocins, which act as nucleases, DNAses, inhibitors of cell wall component production, pore formers, and fermentation products such as short chain fatty acids (SCFAs)) or by effective competition for the utilization of available nutrients and adhesion receptors on the intestinal epithelium, or indirectly via the stimulation of immune defenses [33]. The impairment of colonization resistance may involve the selective elimination of specific species or genera, an overall reduction of microbiota density, or changes in nutrient availability or mucosal physiology (e.g., increased oxygen levels). Specific examples of organisms that have been associated with colonization resistance include lactobacilli, specifically the acidophilus subgroup, which can generate reactive oxygen species. In contrast, the presence of high *Escherichia coli* densities in the gut has been associated with *Salmonella enterica* infection [34,35]. The exact mechanisms by which the intestinal microbiota is able to limit the proliferation of *C. difficile* are still under active investigation, but may involve alteration of the bile acid metabolism and competition for nutrients.

### 3.1. Influence of Bile Acids on C. difficile Spore Germination and Vegetative Growth

**Figure 1:**
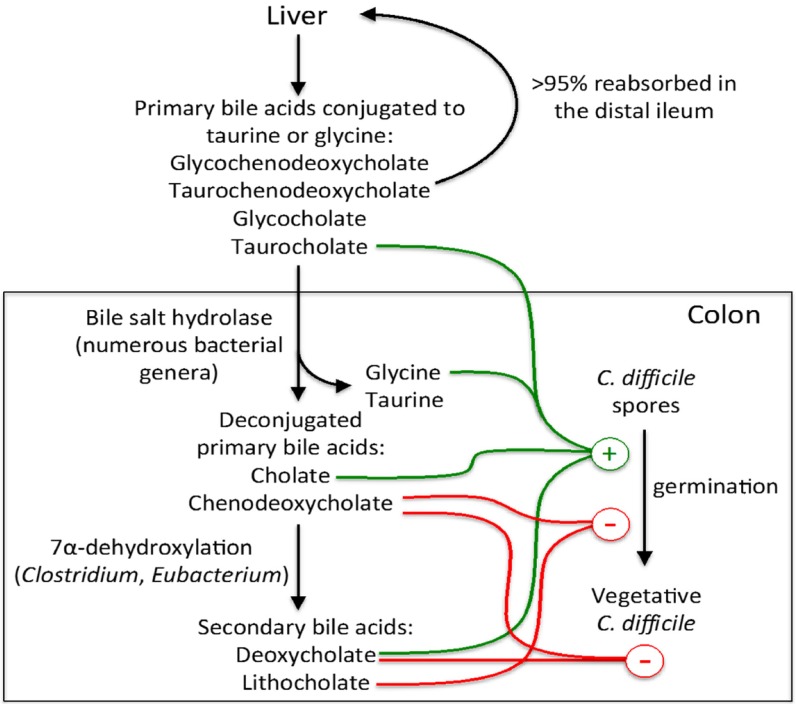
Metabolism of bile acids and their impact on the germination and growth of *C. difficile*. Primary bile acids are synthesized by the liver and excreted in the gastrointestinal tract but the majority are reabsorbed in the distal ileum and returned to the liver via enterohepatic circulation. The fraction of bile acids that escapes enterohepatic circulation passes into the colon where they are metabolized and transformed into secondary bile acids by the gut microbiota. Bile acids and their metabolites can either inhibit or enhance the germination of spores, as well as the growth of vegetative *C. difficile.* The overall proportions of bile acids may determine the clinical outcome of *C. difficile* infection. Figure adapted from [45].

The primary bile acids produced by the human liver consist mainly of cholate and chenodeoxycholate, which are typically conjugated to either taurine (e.g., taurocholate) or glycine (Figure 1). The fraction of secreted bile acids that is not reabsorbed in the distal ileum (<5%) passes into the colon, where they undergo bacterial metabolism [36]. A broad spectrum of intestinal bacteria has the ability to deconjugate primary bile acids and a minor fraction of the microbiota can further metabolize them into secondary bile acids, such as lithocholate and deoxycholate [37,38]. *In vitro* studies have shown that the conjugated and deconjugated forms of cholate, along with the amino acid glycine, act together to induce the germination of *C. difficile* spores, whereas chenodeoxycholate is a potent inhibitor of spore germination [39,40]. The secondary bile acid lithocholate inhibits germination, whereas deoxycholate promotes spore germination but is toxic for vegetative *C. difficile* and therefore inhibits its growth [39,41]. Under normal physiological conditions, chenodeoxycholate competitively inhibits taurocholate-mediated spore germination in the gut and also prevents *C. difficile* vegetative growth, as does deoxycholate [39,40,41]. However, when the intestinal microbiota is disrupted by antibiotics, primary bile acids are no longer transformed into secondary bile acids and an increase in the ratio of cholate to chenodeoxycholate derivatives, due to a more rapid absorption of the latter by the colonic epithelium, may promote the germination and growth of *C. difficile* [41]. Recently, Buffie *et al.* found that *Clostridium scindens*, a bile acid 7α-hydroxylating bacterium, displayed strong inhibition against *C. difficile* in the intestinal microbiota of antibiotic-treated mice and humans [42]. The 7α-hydroxylation activity is involved in the conversion of primary bile acids into secondary bile acids and is possessed by a limited number of intestinal species among the *Clostridium* and *Eubacterium* genera [36]. Administration of *C. scindens* to antibiotic-exposed animals enhanced resistance to CDI in a secondary bile acid dependent manner. Although these findings remain to be validated in human populations, they do lay the groundwork for the elaboration of targeted, microbiome-based interventions to prevent and treat CDI in high-risk individuals.

### 3.2. Competition for Resources

Antibiotics can affect the availability of microbial resources in the gut in various ways. First, as a consequence of reducing total bacterial biomass, antibiotics decrease competition for nutrients and also open up previously unavailable ecological niches. In addition, the lysis of bacteria that are susceptible to antibiotics releases carbon sources that can be consumed by the remaining members the microbial community [20]. Almost three decades ago, Wilson and Perini found that the mucin components *N*-acetylglucosamine and *N*-acetylneuraminic acid (a sialic acid) enhanced *C. difficile* growth *in vitro*. Moreover, the addition of these monosaccharides to a culture of mouse cecal microbiota fully restored the ability of the microbial community to suppress *C. difficile* when transferred to germ-free mice, suggesting that these carbohydrates promote the expansion of specific intestinal bacteria that can efficiently utilize them, thereby outcompeting *C. difficile* [43]. In line with these findings, Ng and colleagues recently showed that under normal conditions, the intestinal microbiota competes for the consumption of sialic acids that are liberated from the mucus lining of the gut. However, when the resident microbiota is suppressed by antimicrobials, sialic acids are left unconsumed and this provides a window of opportunity for *C. difficile* to use these monosaccharides and proliferate [44].

The occupation of *C. difficile*’s ecological niche by non-pathogenic (non-toxigenic) *C. difficile* strains may also prevent toxigenic strains from gaining a foothold in the gut by mediating competitive exclusion. Experimental data from hamster models have shown that colonization of the intestines with a non-toxigenic strain of *C. difficile* before challenge with a toxigenic strain can prevent CDI development [46,47,48] and a phase 2 clinical trial recently reported significantly lower rates of recurrent CDI in patients receiving non-toxigenic *C. difficile* [49].

## 4. Human Gut Microbiota and *C. difficile* Colonization and Infection

Although antimicrobial-induced intestinal microbiota disruptions are well recognized to be a major risk factor for CDI, the specific alterations incurred by the intestinal microbiota and how these alterations lead to subsequent CDI development remain unclear. A growing number of studies have described the microbial alterations associated with *C. difficile* colonization and infection in humans (Table 1). Similar studies have also been performed in animal models and are reviewed elsewhere [50]. Although murine models of CDI are valuable tools to study the interplay between *C. difficile* and the intestinal microbiota, members of the human and murine gut microbiota are phylogenetically distinct. Most investigations have used 16S rRNA gene sequencing to describe the composition of the fecal microbiota in subjects with *C. difficile* [51,52,53,54,55,56,57,58]. A minority of studies obtained fecal samples prior to the actual acquisition of *C. difficile* or CDI diagnosis [54,57,59,60]. The majority of studies included a limited number of subjects; only three out of 13 studies analyzed more than 30 patients with *C. difficile* [53,56,61]. Longitudinal sample collection was performed in only two studies [57,60].

Early studies used bacterial culture and *in situ* hybridization with 16S rRNA probes to compare the composition of the fecal microbiota in children (*n* = 10), young adults (*n* = 7), healthy elderly subjects (*n* = 5) and geriatric patients diagnosed with CDI (*n* = 4) [62,63,64]. The fecal microbiota of CDI patients displayed low bacterial content and species diversity. These patients had a sharp reduction in *Bacteroides*, *Prevotella* and *Bifidobacteria*, and an increase in clostridia, lactobacilli and enterobacteria compared to other subject groups. However, the authors acknowledged that some of these alterations may have been caused by the administration of metronidazole to CDI patients prior to stool collection. Chang *et al.* used 16S rRNA gene clone libraries to compare the fecal communities of four patients with an initial episode of CDI, three patients with recurrent CDI and three healthy control subjects [51]. In patients with an initial episode of CDI and in control subjects, the majority of sequences corresponded to organisms within the Firmicutes and Bacteroidetes divisions, which are the two dominant bacterial phyla in the gut of healthy individuals. In contrast, the composition of the fecal microbiota in patients with recurrent CDI was highly variable and displayed a marked reduction in bacterial richness and diversity. In two out of three patients with recurrent CDI, the fecal communities were dominated by Proteobacteria or Verrucomicrobia, which are normally minor constituents of the gut microbiota.

Prospective studies are required to identify causal microbiome-based biomarkers of CDI risk. However, because diagnostic specimens are often easier to obtain, very few studies have described the composition of the intestinal microbiota in the at-risk period for CDI. The collection of fecal samples prior to CDI development is ideal as it prevents confounding of the results by the effects of the infection itself (diarrhea, gut inflammation) and the effects of CDI treatment (with antibiotics) on the intestinal microbiota. De La Cochetière *et al.* investigated the relationship between dominant members of the intestinal microbiota and subsequent acquisition of *C. difficile* in adult outpatients receiving antimicrobial therapy [60]. Fecal samples obtained prior to the initiation of antimicrobial treatment were analyzed by TGGE of the 16S rRNA gene and the resulting microbial profiles could predict the risk of *C. difficile* acquisition in these subjects. These results support the idea that certain patients may have an existing predisposition to *C. difficile* colonization and infection; their intestinal microbiota may be less resilient to the effects of antibiotics or more permissive to the invasion of *C. difficile*.

**Table 1:** Summary of studies that examined the relationship between the human intestinal microbiota and *C. difficile* colonization or infection.

Subjects	Fecal Samples Collected Prior to *C. difficile* Acquisition or CDI Diagnosis	Longitudinal Sample Collection	Inclusion of Epidemiologic Data in Analyses *	Method of Microbiota Measurement	Findings	Reference
Children (*n* = 10), young adults (*n* = 7), healthy elderly (*n* = 5) and geriatric patients diagnosed with CDI (*n* = 4)	No	No	No	Bacterial culture and *in situ* hybridization with 16S rRNA probes	• In patients with CDI, the bacterial content and species diversity was markedly reduced. • CDI patients had significantly lower levels of *Bacteroides-Porphyromonas-Prevotella* and higher numbers of enterobacteria, clostridia and lactobacilli compared to other subject groups.	[62,63,64]
Patients with an initial episode of CDI (*n* = 4), patients with recurrent CDI (*n* = 3) and healthy subjects (*n* = 3)	No	No	No	16S rRNA gene clone libraries	• The richness and diversity of the gut microbiota was consistently lower in patients with recurrent CDI compared to patients with an initial episode of CDI and healthy subjects. • In patients with recurrent CDI, the composition of the microbiota was highly variable and deviated from the normal predominance of Bacteroidetes and Firmicutes.	[51]
Adult outpatients who acquired *C. difficile* (*n* = 11), or did not acquire *C. difficile* (*n* = 76) during antibiotic treatment	Yes	Yes	Yes	Temporal temperature gradient gel electrophoresis (TGGE) of the 16S rRNA gene (V6–V8 region)	• The TGGE profiles did not cluster based on the presence or absence of *C. difficile*. • TGGE profiles explained 46% (prior antibiotic treatment) and 74.5% (after antibiotic treatment) of the variation associated with *C. difficile* acquisition. • Dominant bands specific for the group of patients who acquired *C. difficile* corresponded to the Lachnospiraceae and Clostridiaceae families and *Eubacterium* and *Ruminococcus* genera.	[60]
Patients with CDI (*n* = 25) and hospitalized controls (*n* = 50)	Yes	No	Yes	16S rRNA microarray	• Probes intensities were higher for Firmicutes, Proteobacteria and Actinobacteria in CDI cases compared to controls, whereas probe intensities for Bacteroidetes were lower. • After epidemiologic factors were controlled for, only Firmicutes and Bacteroidetes remained significantly and independently associated with CDI development.	[59]
Patients with CDI (*n* = 2), asymptomatic *C. difficile* carriers (*n* = 20) and *C. difficile*-negative elderly subjects (*n* = 252)	No	No	No	16S rRNA gene sequencing (V4 region)	• At the family level, Erysipelotrichaceae, Aerococcaceae and Flavobacteriaceae were significantly enriched in the *C. difficile*-positive group, while Enterococcaceae, Leuconostocaceae, Prevotellaceae and Spirochaetaceae were reduced. • The gut microbiota of patients with active CDI was characterized by a dominance of *Parabacteroides* and an absence of *Bifidobacterium* and *Faecalibacterium* spp.	[52]
*C. difficile*-positive patients (*n* = 105), *C. difficile-*negative patients (*n* = 66) and *C. difficile*-negative healthy subjects (*n* = 37)	No	No	No	Denaturing high-pressure liquid chromatography	• Bacterial richness and diversity was significantly lower in patient samples than in healthy subjects. However, bacterial and fungal richness did not differ between *C. difficile*-positive and -negative patient samples. • Among the subset of patients with diarrhea, the frequency of Peptostreptococcaceae, *Ruminococcus bromii* and *Streptococcus*/*Enterococcu*s sp. was higher in *C. difficile*-positive compared to *C. difficile*-negative patients, whereas the frequency of *Bifidobacterium longum* and *Methanobrevibacter smithii* was lower. • The presence of *Bifidobacterium longum* was the most important predictor for the *C. difficile*-negative status.	[61]
Patients with CDI (*n* = 39), patients with *C. difficile*-negative diarrhea (*n* = 36) and healthy controls (*n* = 40)	No	No	No	16S rRNA gene sequencing (V1–V3 region)	• Decrease in microbial diversity and species richness in the CDI and *C. difficile*-negative diarrhea groups. • *Enterococcus*, *Lactobacillus* and *Veillonella* genera, as well as the Gammaproteobacteria class were enriched in CDI patients. • Ruminococcaceae and Lachnospiraceae families as well as butyrate-producing C2 to C4 anaerobic fermenters were significantly depleted in the CDI and *C. difficile*-negative diarrhea groups.	[53]
Patients with CDI (*n* = 25) and hospitalized controls (*n* = 25)	Yes	No	Yes	16S rRNA gene sequencing (V1–V3 and V3–V5 regions)	• Reduced intestinal microbiota diversity in CDI cases. • Sequences corresponding to the phylum Bacteroidetes and to the families Bacteroidaceae and Clostridiales Incertae Sedis XI were depleted in CDI cases compared to controls, whereas sequences corresponding to the family Enterococcaceae were enriched. • In multivariable analyses, antimicrobial use and Clostridiales Incertae Sedis XI remained significantly and independently associated with CDI development.	[54]
Patients with CDI (*n* = 14), asymptomatic *C. difficile* carriers (*n* = 14) patients with *C. difficile*-negative diarrhea (*n* = 16), and patients without diarrhea and *C. difficile*-negative stool (controls, *n* = 15)	No	No	No	Quantitative real-time PCR	• DNA concentrations for *C. difficile* were highest in the CDI group, followed by the asymptomatic carrier group, while the *C. difficile*-negative diarrhea and control groups had very low values • Concentrations of Clostridia sp. and Bacteroidetes were lower in CDI patients and asymptomatic carriers compared to patients with *C. difficile*-negative diarrhea and controls.	[65]
Patients with CDI (*n* = 10), patients under antibiotic therapy who did not develop CDI (*n* = 15) and healthy individuals without antibiotic therapy (*n* = 18)	No	No	No	16S rRNA sequencing from DNA and RNA (V1–V2 region)	• The diversity of the total microbiota (DNA) but not the active subset (RNA) was significantly lower in patients with CDI compared to patients without CDI. • No significant difference in the composition of the total or active gut microbiota between patients with and without CDI. • At the RNA level, *Streptococcus*, *Enhydrobacter* and *Granulicatella* were more predominant in patients with CDI. • At the DNA level, *C. difficile* was more predominant in patients with CDI, while *Adlercreutzia* and *Collinsella* were more predominant in patients without CDI.	[55]
Patients with CDI (*n* = 94), patients with *C. difficile*-negative diarrhea (*n* = 89), and healthy controls (*n* = 155)	No	No	Yes	16S rRNA gene sequencing (V3–V5 region)	• The community structure of CDI cases was similar to that of patients with non-*C. difficile*-associated diarrhea. Diversity was 2-fold lower in patients with *C. difficile*-positive or -negative diarrhea compared to healthy controls. • Patients with CDI were significantly more likely to harbour *Enterococcus*, Enterobacteriaceae, Lachnospiraceae and Erysipelotrichaceae, and significantly less likely to harbour *Bacteroides*, Ruminococcaceae and *Alistipes* than healthy subjects. • Patients with CDI were significantly enriched in Erysipelotrichaceae, Clostridiaceae, *Blautia* and *Clostridium* clusters XI, XIVa, and XVIII compared to patients with *C. difficil*e-negative diarrhea.	[56]
Patients who acquired (*n* = 3) or did not acquire *C. difficile* (*n* = 5) during antibiotic treatment	Yes	Yes	No	16S rRNA gene (V1–V3 region) and metagenomic sequencing	• The richness and diversity of the gut microbiota was lower in *C. difficile*-positive compared to *C. difficile*-negative patients. • In *C. difficile*-positive samples, there was a significant overrepresentation of the genera *Lactobacillus*, *Bacteroides*, *Enterococcus*, *Faecalibacterium*, the family Lachnospiraceae Incertae Sedis and *Clostridium* clusters XIVa and XI, and an underrepresentation of *Roseburia*, *Coprococcus*, *Blautia*, *Subdoligranulum*, *Parabacteroides*, *Oscillibacter*, *Alistipes*, *Butyricicoccus*, *Lactococcus* and *Streptococcus*. • In *C. difficile*-positive samples, there was a significant overrepresentation of “transport and binding proteins”, mainly for “carbohydrates, organic alcohols and acids” and “signal transduction by the phosphotransferase system”, and an underrepresentation of “mobile and extrachromosomal element functions” as well as “aromatic amino acid biosynthesis”.	[57]
Patients with CDI (*n* = 8), asymptomatic *C. difficile* carriers (*n* = 8) and healthy individuals (*n* = 9)	No	No	No	16S rRNA gene sequencing (V3–V4 region)	• Microbial richness and diversity were significantly reduced in CDI patients and asymptomatic carriers compared with healthy controls. • Greater interindividual variation within samples from CDI patients and asymptomatic carriers. • Within Bacteroidetes, there was a significant decrease in the abundance of *Alistipes*, *Bacteroides* and *Prevotella*, while *Parabacteroides* were increased in CDI patients and asymptomatic carriers compared to healthy subjects. • Within Firmicutes, there was a significant decrease in the abundance of *Phascolarctobacterium*, *Roseburia*, *Megamonas*, *Ruminococcus*, *Faecalibacterium* and *Coprococcus*, while *Clostridium* XIVa, *Clostridium sensu strict*, *Enterococcus*, *Veillonella* and *Lactobacillus* were increased in CDI patients and asymptomatic carriers compared to healthy subjects. • Within Proteobacteria, there was a significant decrease in the abundance of *Parasutterella* and *Gemmiger*, while *Escherichia*/*Shigella*, *Klebsiella*, *Haemophilus*, *Pseudomona* and *Bilophila* were increased in CDI patients and asymptomatic carriers compared to healthy subjects	[58]

* In the statistical analysis of microbial features that are associated with *C. difficile* acquisition or CDI development.

Hospitalized patients are at highest risk of developing CDI. However, these subjects are often heterogeneous in terms of underlying disease and medication exposure, all of which can affect the composition of the intestinal microbiota. Only a handful of investigations have accounted for the influence of antimicrobials and other medications in the statistical analysis of microbial profiles associated with CDI [54,56,59,60]. Among them, Manges *et al.* employed 16S rRNA microarrays to examine the composition of the intestinal microbiota in 25 CDI cases and 50 hospitalized controls [59]. Fecal samples were obtained shortly after hospital admission but prior to CDI diagnosis in the cases. At the phylum level, probe intensities for Firmicutes, Proteobacteria and Actinobacteria were higher in patients who developed CDI compared to controls, whereas probe intensities for Bacteroidetes were lower. After adjusting for epidemiologic factors (age, sex and use of fluoroquinolone, cephalosporins or nonsteroidal anti-inflammatory drugs), only a small subset of organisms within the Firmicutes (reduction of Clostridiales Incertae Sedis XI and increases in Enterococcaceae and Lactobacillaceae) and Bacteroidetes (reduction of Porphyromanadaceae) phyla remained significantly and independently associated with CDI development. In a follow-up study [54], the same case-control population was evaluated by high-throughput 16S rRNA sequencing in order to obtain quantitative data and get a broader view of the bacterial taxa that are present in the intestinal tract of patients. In this study, reduced intestinal microbiota diversity was again significantly associated with incipient CDI. In agreement with results from Zhang *et al.* [58], case patients displayed a greater level of heterogeneity in their community membership compared to controls. The abundance of Bacteroidetes (phylum), Bacteroidaceae (family) and Clostridiales Incertae Sedis XI (family) was significantly reduced in CDI cases compared to controls, whereas the abundance of Enterococcaceae (family) was increased. In multivariable analyses, cephalosporin and fluoroquinolone use, as well as a decrease in the proportion of Clostridiales Incertae Sedis XI were significantly and independently associated with CDI development. Using quantitative real-time PCR, Goldberg *et al.* also observed a significant decrease in the abundance of clostridia and Bacteroidetes in patients with active CDI compared to patients without diarrhea and *C. difficile*-negative stools [65].

Rea and colleagues used 16S rRNA gene sequencing to examine the composition of the fecal microbiota in elderly subjects with positive (*n* = 22) or negative (*n* = 252) *C. difficile* culture results [52]. Among culture-positive patients, two displayed CDI symptoms at the time of sampling; the other subjects were asymptomatic *C. difficile* carriers. The bacterial families Erysipelotrichaceae, Aerococcacae and Flavobacteriaceae were significantly increased in *C. difficile* culture-positive compared to culture-negative subjects, while Enterococcaceae, Leuconostocaceae, Prevotellaceae and Spirochaetaceae were reduced. However, the authors noted that these families represented a very small proportion (0.03%–0.05%) of the bacterial populations. In contrast, the fecal microbiota of patients with active CDI was dramatically altered and characterized by a dominance of the genus *Parabacteroides* and an absence of sequences assigned to *Bifidobacterium* and *Faecalibacterium*.

Two recent studies have used 16S rRNA gene sequencing to compare the fecal microbiota of patients with CDI, patients with non-*C. difficile*-associated diarrhea and healthy subjects [53,56]. In both studies, the microbial diversity was markedly lower in patients with *C. difficile*-positive or negative diarrhea than in healthy subjects. In the study by Antharam *et al.*, Ruminococcaceae and Lachnospiraceae, as well as butyrate-producing bacteria (including *Roseburia*, *Faecalibacterium*, *Subdoligranulum*, *Anaerostipes* and *Pseudobutyvibrio*), were significantly depleted in patients with CDI and non-*C. difficile*-associated diarrhea compared to healthy subjects, while lactate-producing bacteria (*Enterococcus* and *Lactobacillus*) were more abundant [53]. In the study by Schubert *et al.*, *Bacteroides*, *Alistipes* and Ruminococcaceae were depleted in patients with CDI and non-*C. difficile*-associated diarrhea compared to healthy controls, while *Enterococcus*, Enterobacteriaceae, Erysipelotrichaceae and members of the Lachnospiraceae family were enriched [56]. These alterations may represent a microbial signature of colonic dysfunction and nosocomial diarrhea.

Skraban and colleagues used denaturing high-pressure liquid chromatography to characterize the bacterial, archaeal and fungal communities in fecal samples from 105 *C. difficile*-positive patients, 66 *C. difficile*-negative patients and 37 *C. difficile*-negative healthy subjects [61]. Bacterial and fungal richness did not differ between *C. difficile*-positive and -negative patients. Among the subset of patients with diarrhea, the frequency of Peptostreptococcaceae, *Ruminococcus bromii* and *Streptococcus*/*Enterococcus* sp. was higher in *C. difficile*-positive compared to *C. difficile*-negative patients, whereas the frequency of *Bifidobacterium longum* and *Methanobrevibacter smithii* was lower.

A limited number of studies have compared the intestinal microbiota of patients with CDI to that of asymptomatic *C. difficile* carriers [52,58,65]. Zhang *et al.* observed that the richness and diversity of the intestinal microbiota was similar for asymptomatic carriers and CDI patients, but the overall structure of the microbial community differed significantly between CDI patients, asymptomatic carriers and healthy individuals [58]. In contrast, Rea and colleagues reported that the microbial profiles were very similar between asymptomatic carriers and culture-negative subjects, suggesting that the normal intestinal microbiota can prevent CDI development [52].

Rather than looking only at the species that are present in the gut (based on 16S rRNA gene amplicons generated from DNA), studies have now begun to address the functional potential of the intestinal microbiome (based on total DNA content) and determine which species are metabolically active (based on RNA content). Knecht *et al.* amplified and sequenced the 16S rRNA gene from genomic DNA or complementary DNA generated from RNA in order to analyse the total and active subsets of the fecal microbiota in patients who developed CDI during antibiotic treatment with β-lactam agents [55]. The diversity was consistently and significantly lower in patients with CDI than in patients without CDI at the DNA (total microbiota) but not at the RNA (active microbiota) level. The overall composition of the total and active gut microbiota was similar for patients with and without CDI. The authors suggested that a reduction in specific, active members of the gut microbiota, rather than a reduction in overall diversity or composition could result in a loss of functional capacities associated with colonization resistance against *C. difficile*. Pérez-Cobas and colleagues used various intra- and inter-individual comparisons of *C. difficile*-positive and -negative samples in order to identify bacterial taxa and metabolic functions involved in colonization resistance against *C. difficile* [57]. Members of the Clostridiales order (*Ruminococcus*, *Subdoligranulum*, *Oscillibacter*, *Gemmiger* and *Anaerovorax*), as well as genes involved in aromatic amino acid biosynthesis, endospore formation, polyamine biosynthesis and stress response mechanisms were identified as potential species and functions mediating protection against *C. difficile* colonization. These metabolic functions may also mediate colonization resistance indirectly, through modulation of the host immune response.

In summary, most studies have observed a decrease in the microbial richness and diversity of the intestinal microbiota in patients with CDI, including a study that collected fecal samples prior to CDI development [54], suggesting that this may be a predisposing factor for CDI. Bacterial groups that were commonly associated with *C. difficile* colonization or infection in reviewed studies are shown in Table 2. Enterobacteria and enterococci are opportunistic microorganisms that can, like *C. difficile*, exploit the reduced biodiversity of the intestinal ecosystem to expand their populations. This idea is consistent with studies showing increased levels of enterococci in the gut following treatment with extended-spectrum antimicrobial agents [66,67]. Lawley and colleagues also found that antibiotic treatment of mice asymptomatically colonized with *C. difficile* resulted in a dramatic reduction in intestinal microbial diversity accompanied by an expansion of *Escherichia coli* and enterococci, which triggered the overgrowth of *C. difficile* [68]. A depletion of Bacteroidetes (including *Bacteroides* and *Prevotella*) is frequently observed in patients who develop *C. difficile* colonization or infection. Animal models have shown that *Bacteroides* play an immunomodulatory role in inflammatory processes of the gut, thereby preventing the development of experimental colitis [69]. Similarly, Ferreira *et al.* have suggested that Bacteroidetes may confer resistance to infectious colitis by protecting against pathogen-mediated intestinal inflammation [70]. Human studies have reported mixed results regarding the association between intestinal Clostridiales and CDI; some have reported an increase in the abundance of clostridia [56,60,62,63,64], while others observed a decrease [54,65]. The Clostridial species that live in the gut probably utilize the same limiting resources as *C. difficile* and may therefore have the ability to outcompete toxigenic *C. difficile* in a similar way as non-toxigenic *C. difficile* does. Antibiotics likely deplete these indigenous clostridial populations and may open up a previously unavailable ecological niche that allows the proliferation of pathogenic *C. difficile* in the gut. *Roseburia* and *Coprococcus* are important members of the gut microbiota that are involved in the production of butyrate [71]. Butyrate is the preferred energy source of colonocytes and plays a major role in the regulation of colonocyte proliferation and differentiation and the maintenance of intestinal epithelium integrity [72]. It can protect the host from infection by enhancing colonic defense barriers through the stimulation of mucin and antimicrobial peptide production [72,73]. This SCFA was shown to inhibit *C. difficile* growth *in vitro* [74,75], has well-documented anti-inflammatory effects in the gut and is thought to protect against colitis [72,73,76]. Therefore, a depletion of butyrate producers may alter host defenses against *C. difficile* and increase the susceptibility to CDI.

**Table 2:** Fecal bacterial groups that are frequently associated with *C. difficile* colonization or infection in humans.

Increased Abundance	Decreased Abundance
*Enterococcus*	Bacteroidetes (*Bacteroides* and *Prevotella*)
*Lactobacillus*	*Bifidobacterium*
Erysipelotrichaceae	*Alistipes*
Proteobacteria (Enterobacteriaceae)	*Roseburia*
*Clostridium* clusters XI and XIVa	*Coprococcus*
*Veillonella*	

## 5. Fecal Microbiota Transplantation

FMT (also known as fecal bacteriotherapy) is a therapeutic procedure that involves the instillation of stool suspensions from a healthy donor into the gastrointestinal tract of patients infected with *C. difficile*. This biotherapy represents a paradigm shift in the approach to CDI treatment, as it focuses on the restoration of the normal gut microbiota rather than on the eradication of the pathogen with antibiotics. The procedure was described for the first time in the modern medical literature in 1958, when Eiseman and co-authors reported the resolution of pseudomembranous colitis in four patients within hours after the administration of fecal enemas [77]. Since then, more than 500 patients were reported to have undergone FMT worldwide [78]. Despite variations in protocols for the preparation of fecal material and routes of administration, all studies to date have shown that FMT is a safe and highly effective treatment for recurrent CDI, achieving cumulative cure rates greater than 90% [78,79]. In a recent randomized controlled study, the trial was terminated after interim analysis, as resolution rates for recurrent CDI were significantly higher in patients who received an infusion of donor feces through a nasoduodenal tube (81%) than in patients who received a standard vancomycin regimen alone (31%) or in combination with a bowel lavage (23%) [18]. The procedure is inexpensive and typically produces a clinical response within hours to days after transplantation [16]. The restoration of the intestinal microbiota, and therefore colonization resistance, is thought to be responsible for the eradication of recurrent CDI in patients who undergo FMT. However, other substances present in feces, such as bile acids and immune cells or mediators, may explain the higher success rate of FMT compared to probiotic therapy.

### 5.1. Recovery of the Intestinal Microbiota after FMT

Recent studies have described the recovery of gut bacterial populations after FMT and showed that this procedure has a dramatic and persistent impact on the composition of the intestinal microbiota. Prior to FMT, the fecal microbiota of patients suffering from recurrent CDI typically displays reduced diversity, is deficient in members of the Firmicutes and Bacteroidetes phyla, and is often dominated by Proteobacteria [18,51,80,81,82]. Resolution of recurrent CDI after FMT is associated with a restoration of bacterial diversity and the recovery of Firmicutes and Bacteroidetes populations in the intestinal microbiota [18,80,82]. Within days after the FMT procedure, the fecal microbiota profile of the recipients closely resembled that of the healthy donors, suggesting successful engraftment of the transferred microbial community, although the transplanted microbiota eventually adapted to its new host and diverged from the donor over time [80,81]. Khoruts and colleagues assessed the composition of the fecal microbiota in a single patient with relapsing CDI who underwent FMT via colonoscopy [81]. After the procedure, the patient’s symptoms promptly resolved and her gut microbiota was dominated by *Bacteroides* and butyrate-producing bacteria, including Ruminococcaceae and *Anaerostipes*. In the largest case series performed so far, Shahinas and colleagues explored bacterial populations in pre- and post-transplantation stool samples obtained from 6 patients with recurrent CDI [82]. The fecal samples collected prior to FMT or after a FMT failure were dominated by Proteobacteria, while the fecal microbiota of donors and successful FMT recipients had higher levels of Bacteroidetes and Firmicutes. After successful FMT, there were marked increases in *Bacteroides*, *Lactobacillus*, *Dorea*, *Roseburia* and *Faecalibacterium* with corresponding decreases in Enterobacteriaceae and unclassified Proteobacteria. In the first randomized controlled trial of FMT, the recipients’ microbiota showed an increase in Bacteroidetes and *Clostridium* clusters IV and XIVa, combined with a decrease in Proteobacteria [18].

### 5.2. Elaboration of Bacterial Mixtures as a Stool Substitute for FMT

Given its low cost, safety and effectiveness, FMT is currently being integrated into mainstream clinical practice as the treatment of choice for recurrent CDI. Efforts are also being made to identify the specific microorganisms in stool that are able to resolve recurrent CDI. Defined communities of bacteria have been shown to be effective in preventing recurrent CDI in patients and animal models [83,84,85]. More than two decades ago, Tvede and Rask-Madsen instilled a mixture of 10 bacterial species (including *Clostridium*, *Bacteroides*, *Escherichia coli*, *Enterococcus faecalis* and *Blautia producta*) into the rectum of five patients with recurrent CDI, which resulted in the eradication of *C. difficile* and its toxins from the stool specimens, the restoration of normal bowel function within 24 h and the disappearance of abdominal symptoms [84]. The patient samples remained negative for *C. difficile* and its toxins up to six months after treatment. The authors also noted that although *Bacteroides* sp. were not present in the patients’ specimens prior to treatment, this genus persistently colonized the intestines of all patients after administration of the bacterial mixture. More recently, Petrof and colleagues introduced a combination of 33 intestinal bacteria (a stool substitute called “RePOOPulate”) purified from the feces of a healthy donor into the bowels of two patients suffering from recurrent CDI [85]. Within two to three days after treatment, both patients reverted to normal bowel patterns and remained symptom-free for at least six months after transplantation despite exposure to additional antibiotics. Fecal microbiota analysis revealed that while sequences corresponding to the bacteria in the stool substitute were rare in the pre-treatment stool samples, they became transiently abundant and still constituted more than 25% of the sequences up to six months after treatment, demonstrating that some of the administered bacteria can stably colonize the recipients’ intestinal tracts. The efficacy of transplantation with the bacterial preparations described above, as well as with fecal samples obtained from a variety of donors with different microbiota profiles suggests that several combinations of bacteria are able to halt CDI recurrences. Moreover, a combination of a few species appears sufficient to outcompete *C. difficile* and restore gut homeostasis. Such bacterial mixtures may eventually replace the use of feces in the treatment of recurrent CDI.

## 6. Conclusions

Although several studies have described the composition of the intestinal microbiota in subjects who develop *C. difficile* colonization or infection, the specific alterations associated with loss of colonization resistance and subsequent development of CDI remain unclear. Animal models have begun to elucidate the key microbiota species and mechanisms involved in colonization resistance against *C. difficile*. Prospective studies describing the functional disturbances of the intestinal microbiome that precedes CDI developement are needed and will benefit from the increasing accessibility of metagenomic, metatranscriptomic, metaproteomic and metametabolomic approaches. The field will also profit from a growing number of experimental studies and clinical trials aimed at improving the prevention and treatment of CDI, and augmenting our understanding of how antimicrobial-induced gut microbiota disruptions allow *C. difficile* to overcome the defensive mechanisms of colonization resistance.
